# The Role of Reading Fluency in Children’s Text Comprehension

**DOI:** 10.3389/fpsyg.2015.01810

**Published:** 2015-11-27

**Authors:** Marta Álvarez-Cañizo, Paz Suárez-Coalla, Fernando Cuetos

**Affiliations:** Department of Psychology, University of OviedoAsturias, Spain

**Keywords:** prosody, text reading, reading comprehension, Spanish, children

## Abstract

Understanding a written text requires some higher cognitive abilities that not all children have. Some children have these abilities, since they understand oral texts; however, they have difficulties with written texts, probably due to problems in reading fluency. The aim of this study was to determine which aspects of reading fluency are related to reading comprehension. Four expositive texts, two written and two read by the evaluator, were presented to a sample of 103 primary school children (third and sixth grade). Each text was followed by four comprehension questions. From this sample we selected two groups of participants in each grade, 10 with good results in comprehension of oral and written texts, and 10 with good results in oral and poor in written comprehension. These 40 subjects were asked to read aloud a new text while they were recorded. Using Praat software some prosodic parameters were measured, such as pausing and reading rate (number and duration of the pauses and utterances), pitch and intensity changes and duration in declarative, exclamatory, and interrogative sentences and also errors and duration in words by frequency and stress. We compared the results of both groups with ANOVAs. The results showed that children with less reading comprehension made more inappropriate pauses and also intersentential pauses before comma than the other group and made more mistakes in content words; significant differences were also found in the final declination of pitch in declarative sentences and in the F0 range in interrogative ones. These results confirm that reading comprehension problems in children are related to a lack in the development of a good reading fluency.

## Introduction

The difficulty understanding written texts is a major cause of school failure because it requires some cognitive abilities, such as previous knowledge activation, inference performance, mental models building, etc., which not all children have. But some children, despite having these skills, fail in understanding texts. Following the Simple View of Reading (Hoover and Gough, 1990), linguistic comprehension and word recognition are needed to achieve reading comprehension. Besides, fluency could facilitate reading comprehension because it frees resources for understanding (Adlof et al., 2006). Therefore, there could be several causes of this poor comprehension; one of them could be that they have not developed a good reading fluency nor have poor decoding skills. Fluent reading involves accuracy, speed and good expression (National Institute of Child Health, and Human Development, 2000). These three characteristics depend on several cognitive processes and are usually achieved in that order, although overlapping. There are some evidences about the relationship between text reading fluency and reading comprehension, Kim and Wagner (2015) showed that the role of text reading fluency walks together the reading comprehension improvement.

Text reading accuracy is one of the more decisive factors in reading comprehension. Thus, if a child makes many mistakes he cannot understand what he is reading. Moreover, there are some words that are more difficult to read, such as long words (Muncer et al., 2014), low frequency words (Fischer-Baum et al., 2014), words with few orthographic neighbors (Laxon et al., 2002), late age of acquisition words (Cuetos and Barbón, 2006; Monaghan and Ellis, 2010; Davies et al., 2014) or words with complex syllabic structure (Taft, 1979; Rouibah et al., 2000). These kinds of words are often read with less accuracy, and that could affect comprehension.

Speed is also an important part of the reading process. Perfetti (1985) in his Verbal Efficiency Theory states that readers who lack efficient word identification procedures are at risk for comprehension failure. If readers are quick and accurate in identifying words, they will have more attentional resources to devote to understanding what they are reading. Therefore, slowness is also an additional problem, as it consumes working memory and, thus, prevents the reader from thinking about the text while reading. Consequently, slow reading especially affects long sentences, because when the reader finishes with the last words of the sentence, he has already forgotten the first ones.

Another important process of reading fluency is expressiveness, or prosody. Some authors defined fluency as the ability to project natural pitch, stress and juncture of spoken words or written text automatically and at a natural rate (Richards, 2000), considering equal prosody and fluency. Besides, other authors consider that fluency is related, not only with appropriate prosody, but with a deep reading understanding (Rasinski, 2004; Ravid and Mashraki, 2007; Hudson et al., unpublished manuscript), prosody becomes a link between fluency and comprehension (Kuhn and Stahl, 2003). However, the direction of the relationship between prosody and comprehension is not clear.

There are some prosodic markers that are indicative of the reader’s ability (Dowhower, 1991), such as pausal intrusions, final lengthening in sentences, terminal intonation contours, or stress. Schwanenflugel et al. (2004) purposed five prosodic features: the duration and the variation of appropriate and inappropriate pauses, the pitch sentence and the final declination of pitch in sentence. Good readers usually made fewer and shorter pauses within and between sentences, while less skilled children paused often (Schwanenflugel et al., 2004; Miller and Schwanenflugel, 2006; Benjamin and Schwanenflugel, 2010). Similar results have also been found in studies with adults (Binder et al., 2013), since those with low literacy skills made more word and sentence intrusions compared to the skilled adult readers. Thus, these readers made a higher number of inappropriate pauses while reading and for longer durations.

Moreover, Clay and Imlach (1971), from their study with 7-years-old children, suggested that good readers made not only fewer and shorter pauses, but also had a specific contour pitch in declarative sentences when reading. Similar results were found by Miller and Schwanenflugel (2006, 2008), as they reported that adequate pitches and better abilities to decode are related. In addition, children who used larger pitch changes and larger end-sentence declinations in reading performed better on reading comprehension than children who used these prosodic features to a lesser extent (Benjamin and Schwanenflugel, 2010).

In transparent orthographic systems, like the Spanish language, children soon get a high level of accuracy, because it is easier to automate the conversion of graphemes into phonemes; this allows children, after the first year of reading learning, a reading accuracy of 95% of words, contrary to opaque languages where the accuracy is about 35% of the words read (Seymour et al., 2003). However, it is possible that early accuracy leads to neglectful reading, and consequently children take a long time to acquire reading fluency. Besides, the prosody is less worked in schools, maybe because of the difficulty of quantifying.

There are scales to measure some specific features of prosody, such as the Dynamic Indicators of Basic Early Literacy Skills (DIBELS; Kaminski and Good, 1998), which was validated for assessing reading fluency in DIBELS ORF (DIBELS Oral Reading Fluency; Kame’enui et al., 2006). This scale measures speed, accuracy and pauses when children read a text for 1 min. It is usually used as a measure of the progress of students, who may be at risk for difficulties in future reading comprehension in the educational field. Petscher and Kim (2011) used DIBELS ORF to assess children from different grades; the results did not validate the use of oral reading fluency as the sole measuring of children’s reading. Another scale, the Multidimensional Fluency Scale (Rasinski et al., 2009), consists of three subscales to assess phrasing and expression, accuracy and smoothness and pacing. Finally, another scale (Klauda and Guthrie, 2008) assesses several prosodic dimensions, such as expressiveness, phrasing, pace or smoothness. In Spanish, González-Trujillo et al. (2014) created the Reading Fluency Scale in Spanish, based on the Multidimensional Fluency Scale, which consists of the assessment of speed, accuracy and several prosodic features (i.e., volume, intonation, pauses, phrasing, and the reading quality). Children from different grades were assessed using this scale (Calet et al., 2015) and their results showed that also in Spanish, prosodic reading predicts reading comprehension, but depending on the scholar’s grade. These scales are very useful in the educational field, but they have some subjectivity.

Today, thanks to programs like Praat (Boersma and Weenink, 2015), it is possible to measure the components of prosody by analyzing the acoustic wave. This is an objective measure of the prosodic features. This software is a tool for phonetic analysis of speech to analyze prosodic aspects such as frequency, intensity or duration.

The aim of this study was to determine which aspects of reading fluency are related to understanding, that is we are interested in the mechanic aspects of reading that could be related to reading comprehension. In this way, several prosodic features, such as pitch, intensity, pauses, duration of syllables and utterances, were collected using Praat software. Besides, words with different lexical frequency and stress were included. A group of children from third and sixth grade with low written comprehension was compared with a group of children with good written comprehension to deal with the objective.

## Materials and Methods

### Participants

A total of 103 primary school children (58 females) participated in this study. Forty-six were attending third grade (*M_age_* = 8.86, *SD* = 0.39) and fifty-seven were sixth grade students (*M_age_* = 11.89, *SD* = 0.27) in a monolingual school which served children from early childhood (3 years) to high school (17 years). They all had Spanish as their first language and the school served a broadly typical catchment area with the majority children coming from mid-income backgrounds. None of them had developmental, behavioral, or cognitive problems and they also attend school regularly.

This group of children received four expositive texts from the PROLEC-R test (Cuetos et al., 2007), firstly two presented in an oral way (i.e., “El ratel” [“Honey badger”] and “Los vikingos” [“Vikings”]), and secondly two in a written format (i.e., “Los indios apaches” [“Apache Indians”] and “Los okapis” [“Okapis”]). The texts were presented in the same order for all the children. Each text was followed by four questions (two inferential and two literals) in order to measure their oral and written comprehension (see **Table 1** for the main means). We selected the children with better results in oral comprehension, with scores between 6 and 8 points out of eight in the oral comprehension texts, in order to ensure that they had the necessary cognitive abilities needed for comprehension. These children were divided into two groups according to their level of reading comprehension, high reading comprehension group when written comprehension results were similar to the above, and low reading comprehension group when they were about three or four points out of eight. In this way two groups of 10 participants in each grade were selected (see **Table 2**). After this selection we had 40 children: 20 children (13 females) with good oral and written comprehension (“Good comprehension”) and 20 (12 females) with only good oral comprehension (“Poor comprehension”). Therefore, there were no significant differences between groups in oral comprehension scores [*t*(19) = 0.79, *p* = 0.48], while there were in written comprehension scores [*t*(19) = 10.18, *p* < 0.001]. These two final groups were considered the experimental groups, which participated in the second part of the study (as described below). Selected children were also assessed with the reading of words and pseudowords subtests of the PROLEC-R test in order to ensure that everyone had an adequate reading level by age and scholar grade. The poor comprehension group had lower scores than the good comprehension one, but the differences were not significant in both reading of words [*t*(19) = 0.78, *p* = 0.45] and reading of pseudowords [*t*(19) = 2.07, *p* = 0.052] subtests. However, there are significant differences between both groups of third grade in reading of pseudowords subtest [*t*(9) = 2.9, *p* = 0.02], while there are not in sixth grade [*t*(9) = 0.6, *p* = 0.54]. Regarding reading words subtest, there were not significant differences in third grade [*t*(9) = 0.37, *p* = 0.72] nor sixth grade [*t*(9) = 0.73, *p* = 0.49]. PROLEC-R is a standardized battery for the assessment of reading in Spanish children between 6 and 12 years. The reading of words subtest consists of a list of 40 real words, with two or three syllables. In the reading pseudowords subtest children have to read a list of 40 pseudowords, paired by number of syllables, syllabic structure and initial letters with the words list. Children have to read the words and pseudowords aloud; the measurements taken are the number of errors and the time they spent reading each list.

**Table 1:** Results in comprehension questions of the initial participants.

	Third grade	Sixth grade
	*M (SD)*	*M (SD)*
Oral comprehension accuracy (out of eight)	4.3 (1.79)	5.3 (1.66)
Written comprehension accuracy (out of eight)	3.9 (1.78)	5.9 (1.53)

**Table 2:** Results in PROLEC-R test and comprehension questions of texts.

	Third grade		Sixth grade	
	Good comprehension	Poor comprehension	Good comprehension	Poor comprehension
	*M (SD)*	*M (SD)*	*M (SD)*	*M (SD)*
Oral comprehension accuracy (out of eight)	5.9 (0.9)	6 (0.7)	6.6 (0.7)	6.8 (0.4)
Written comprehension accuracy (out of eight)	6.1 (0.7)	3 (1.1)	6.9 (0.7)	4.8 (0.9)
Words accuracy (out of 40)	38.9 (0.9)	38.6 (2.1)	39.5 (1.1)	39 (1.6)
Pseudowords accuracy (out of 40)	32.8 (3.5)	29.4 (3.5)	35.2 (2.9)	34 (3.6)

This research was approved by the Ethics Committee of the Psychology Department of the University of Oviedo. Before starting the experimental tasks, the children’s parents received pertinent information about the purpose of the study, the tasks and their duration. Then, written informed consent was received from the parents of participants.

### Material

A narrative text composed by 306 words, titled “El Gigante Egoísta [The Selfish Giant]” (an adaptation of the story by Oscar Wilde), was used. The text was created including declarative (i.e., “Todos eran amigos de Pablo” [“All of them are Pablo’s friends”], “Una mañana el Gigante oyó el trino de un pájaro” [“One morning the Giant heard a bird’s warble”]), exclamatory (i.e., “¡Qué feliz soy aquí!” [“How happy I am here!”], “¡Por fin ha llegado la primavera!” [“Spring has come at last!”]) and interrogative sentences (i.e., “¿Por qué tarda tanto en llegar la primavera?” [“Why does it take so long to get spring?”] and “¿Qué está pasando en mi jardín?” [“What is happening in my garden?”]). It also included eight low frequency words (*M_lexicalfrequency_* = 13.5; e.g., magnolia [magnolia], secuoya [secoya], subyugado [charmed]), half of them repeated twice, once at the beginning and once at the middle of the text; besides we incorporated 10 words stressed in the penultimate syllable (e.g., palomas [doves], hormigas [ants], tamarindos [tamarinds], estorninos [starlings]), and 10 words stressed in the antepenultimate syllable (e.g., bárbaro [barbarous], mágico [magical], ánfora [amphora], pelícanos [pelicans]), half with low (*M_lexical frequency_* = 5.9) and half with high lexical frequency (*M_lexical frequency_* = 143.9). The lexical frequency was obtained from the database of Martínez and García (2004), who acquired their frequency from a sample of children’s books.

The text was presented on a piece of paper (Times New Roman, 12 point font, double spaced) and the participants had to read it aloud individually in a quiet room. The reading was recorded by an H4n voice recorder and an Ht2-P Audix headset dynamic microphone. Audio recordings were processed oﬄine using Praat software.

### General Assessments

From the .wav files recorded we collected several prosodic parameters using Praat software. First, we analyzed some characteristics of the whole text, and then we extracted six sentences, two declarative, two exclamatory and two interrogative sentences, in order to evaluate different parameters. Finally, we selected eight low frequency words, half of them repeated twice in the text, and eight words with different stresses (on the penultimate and on the antepenultimate syllable) and frequency (high and low).

From the whole text we considered the number of reading mistakes in the content and function words, and the number and duration of intersentential pauses (before commas and full stops) and inappropriate pauses (pauses made in not corresponding places). Also the total pause duration and the total pronunciation time (reading time between pauses) were collected.

Secondly, from the target sentences several measures were used:

Fundamental frequency (F0) measures:

• Range of the first peak (Hz): distance between minimum F0 at the beginning of the sentence and the first peak of F0.
• Total range (Hz): distance between the minimum and the maximum F0 of the sentence.
• Pitch change between different points:
  ◦ From the beginning of the sentence and the first peak (Hz).
  ◦ From the first peak to the end of the sentence (Hz).
  ◦ From the last peak to the end of the sentence (Hz).
  ◦ Between the last syllable and the previous (Hz).
• Slope (Hz/s): declination of the F0 from the first peak to the end of the sentence by time.
  Duration measure:
• Phrase-final lengthening (ms): duration of the last syllable of the sentence in comparison with the previous.
  Intensity measure:
• Intensity change at the end of the sentence (dB): comparison between the intensity of the last syllable with the previous.

With regards to the target words, the number of errors and mean time of reading were measured. In the case of the words with different stresses, we classified the errors in misreading words and changes in the stress place.

## Results

We compared the results of the different parameters of both groups and grades with ANOVAs using SPSS software. Therefore, we used the results of the measurements described above as dependent variables and the grade (third vs. sixth) and group (poor vs. good comprehension) as the independent variables. We named “Poor comprehension group” as the children with better oral than reading comprehension, and “Good comprehension group” as the children with similar oral and reading comprehension. We made ANOVAs with each dependent variable in order to check which the significant effects were. Only those significant are presented here to facilitate the understanding of the results.

### Text Analyses

We found significant differences by group in the number of reading errors made in content words [*F*(1,36) = 7.85, *p* = 0.008] and in the number of inappropriate pauses [*F*(1,3) = 4.18, *p* = 0.048]. Also found was an interaction between group and grade in the number of intersentential pauses before commas [*F*(1,36) = 4.1, *p* = 0.045]. We performed *post hoc* comparisons using Tukey HSD test and we found that the significant differences in these triple interaction were between third and sixth grades within the poor comprehension group (*p* = 0.024). See **Table 3** with the means and SD of these significant effects.

**Table 3:** Mean and SD of the principal significant effects in the text analyses.

Significant effect		Poor comprehension	Good comprehension
		*M (SD)*	*M (SD)*
Number of reading errors in content words		9.9 (0.65)	7.5 (0.65)
Number of inappropriate pauses		70.5 (4.3)	58 (4.3)
Number of intersentential pauses before comma	Third grade	6.5 (1.8)	5.2 (2.6)
	Sixth grade	4.7 (1.4)	5.8 (1.2)

### Sentences Analyses

**FIGURE 1:**
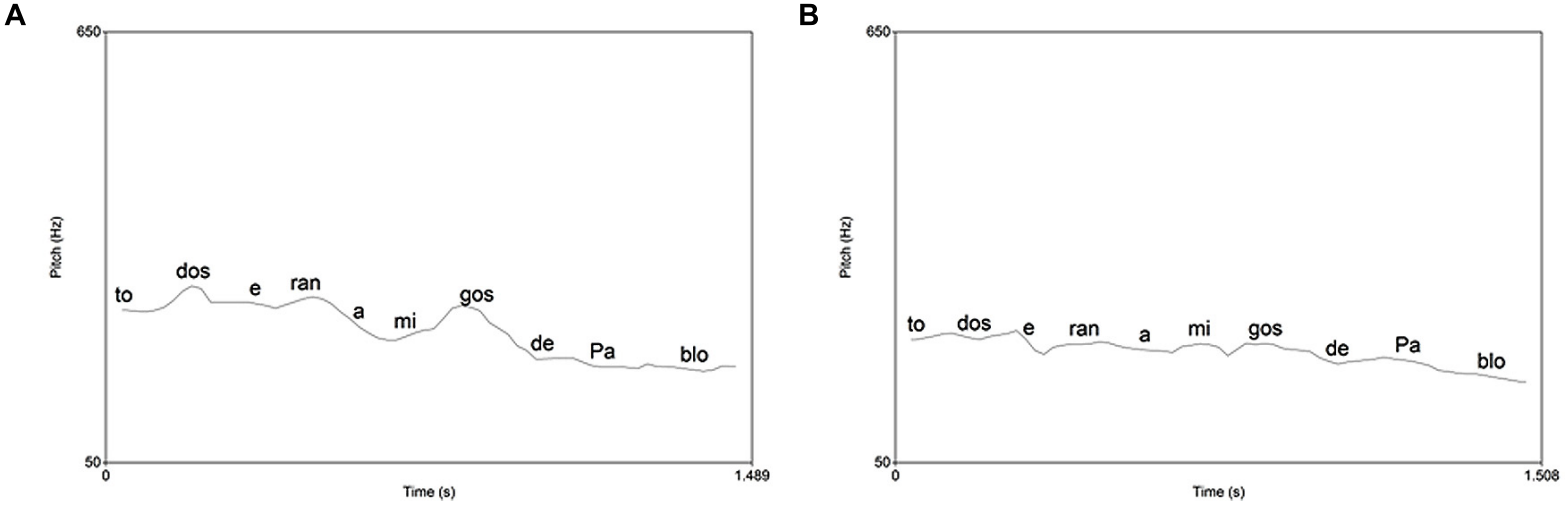
**Pitch contour of a declarative sentence in the good comprehension group **(A)** and the poor comprehension group (B)**.

**FIGURE 2:**
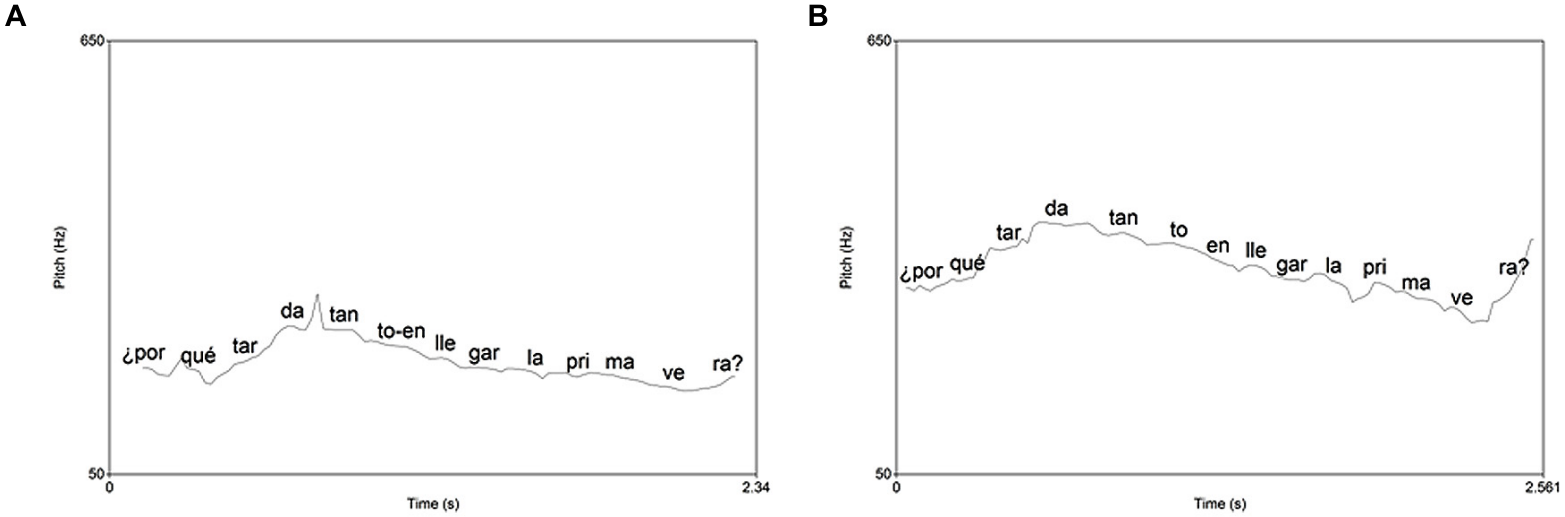
**Pitch contour of an interrogative sentence in the good comprehension group **(A)** and the poor comprehension group (B)**.

We discarded for the analysis all the sentences read incorrectly, which was usually regressions in the reading, around 9%. We analyzed with SPSS software the results from the Praat analysis comparing the two groups and grades using ANOVAs. We found a significant group effect in the fundamental frequency of syllables in declarative sentences [*F*(1,34) = 4.6, *p* = 0.038]. In the frequency range of interrogative sentences the interaction between group and grade was also significant [*F*(1,35) = 6.9, *p* = 0.012]. We made *post hoc* comparisons using the Tukey test, showing that the significant differences were between the two groups of third grade (*p* = 0.048). See **Figures 1** and **2** as examples of the effects found in these analyses and **Table 4** with the means and SD.

**Table 4:** Mean and SD of the principal significant effects in the sentences analyses.

Significant effect		Poor comprehension	Good comprehension
		*M (SD)*	*M (SD)*
F0 of syllables in declarative sentences	Other syllables	221 (7.8)	234 (7.4)
	Last syllable	259 (6.9)	251 (6.5)
F0 range of interrogative sentences	Third grade	196 (61)	157 (34.9)
	Sixth grade	148 (30.8)	194 (63.6)

### Words Analyses

Firstly, we analyzed the low frequency words repeated and non-repeated, discarding those misread. SPSS software was used to conduct ANOVAs for comparing groups and grades. No significant effect by groups was found (*p* > 0.05).

Secondly, an ANOVA was performed with the words with different stress and frequency. There was a significant interaction between the number of errors in the words with different lexical frequency by group [*F*(1,36) = 6.4, *p* = 0.016]. Also an interaction of the mean time for reading high frequency words stressed on the penultimate syllable and group, and grade was found [*F*(1,36) = 5.7, *p* = 0.022]. *Post hoc* comparisons using the Tukey HSD test indicated that the mean reading time of children with poor comprehension from third grade was significantly different from the same group of sixth grade (*p* < 0.001). Finally, a similar interaction of the mean time for reading high frequency words stressed on the antepenultimate syllable and group, and grade was found [*F*(1,36) = 4.6, *p* = 0.038]. The above *post hoc* comparisons also showed that the significant differences were between poor reading comprehension group children in third and sixth grade (*p* = 0.001). See **Table 5** for the means and SD.

**Table 5:** Mean and SD of the primary significant effects in the word analyses.

Significant effect		Poor comprehension	Good comprehension
		*M (SD)*	*M (SD)*
Number of mistakes	High frequency	0.3 (0.07)	0.25 (0.07)
	Low frequency	1.57 (1.4)	1.02 (1.4)
Mean time for reading HF words stressed on the penultimate syllable	Third grade	0.67 (0.06)	0.62 (0.05)
	Sixth grade	0.54 (0.05)	0.57 (0.04)
Mean time for reading HF words stressed on the antepenultimate syllable	Third grade	0.58 (0.09)	0.51 (0.05)
	Sixth grade	0.46 (0.05)	0.48 (0.05)

## Discussion

The aim of this study was to investigate the relationship between comprehension and prosody, both as a part of reading fluency (Rasinski, 2004; Ravid and Mashraki, 2007; Hudson et al., unpublished manuscript). To achieve this objective, we selected two groups of children according to their level of reading comprehension in third and sixth grade. The task consisted of reading aloud a text containing several sentence types and words with different characteristics.

Our results revealed that reading accuracy and reading comprehension are related, as we can see that children with poor reading comprehension made more mistakes in content words than children with good reading comprehension. Also this group was more affected by lexical frequency, since they made a higher number of mistakes in the low frequency words, independently of the stress. A low reading accuracy make children to more misread words and, as Perfetti (1985) stated, readers who fail in word identification will be poorer comprehenders, because of working memory. There are two points of view about the relationship between working memory and reading comprehension, as we could see in the review of this issue made by Van Dyke and Shankweiler (2013). The first one believes that working memory is limited and when it is busy with the decoding not attends to comprehension. The other one related reading comprehension also with high-quality lexical representations. Nevertheless, we have seen that the children with a worse comprehension made more mistakes while reading and also had low scores in the initial subtests of PROLEC-R. Therefore, we could think that one of the causes for poor comprehension could be a low decoding skill that does not allow children to read accurately; as saying by the Simple View of Reading (Hoover and Gough, 1990) decoding is a necessary skill for reading comprehension. It seems clear that children with more reading mistakes show more difficulties to understand when reading because the errors do not allow them process the whole text, but only a part.

On the other hand, better comprehenders had lower reading times in high frequency words with stress on the penultimate and on the antepenultimate syllables than in the same low frequency words. Besides, we found significant differences between grades in the group with poor comprehension; the third-grade children had higher reading times than the sixth-grade children. That did not occur within the group with good reading comprehension, where there was no significant difference between two grades. It could be due to lexical frequency having more weight than the stress place for children with lower reading skills, and as a consequence they read the words with high lexical frequency faster and more accurately. However, this is not what usually happens, as there is a clear tendency to read the words as stressed on the penultimate syllables and make more mistakes when they are stressed on the last and antepenultimate syllable (Gutiérrez-Palma et al., 1998).

In addition, there is a relationship between pausal intrusions and the understanding of the text, since children with a poor reading comprehension made more inappropriate pauses. This was reported in other studies where children with higher fluency made fewer ungrammatical pauses (Miller and Schwanenflugel, 2006; Benjamin and Schwanenflugel, 2010; Alves et al., 2014); the same relationship appears in adults (Binder et al., 2013), where those with low literacy skills made more sentence intrusions compared to the skilled adult readers. Thus, readers with better decoding and word reading skills paused less frequently than readers who had poorer decoding and word reading skills. In addition, readers who experience fewer word and sentence intrusions had better comprehension abilities. Making many pauses involves an increase of the reading time, which would require greater working memory, as Perfetti (1985) considered that more work memory requirement reduced the number of available resources for understanding. That is, when a reader makes a higher number of pauses more working memory is needed and this means less understanding. This greater number of inappropriate pauses made by those children with poor understanding may be due to a low decoding skill. This group of children seems to have difficulty to decode rightly unfamiliar words (i.e., pseudowords and low frequency words), and this may make them stop inappropriately more often in the middle of the words or before unknown words. But not only were the inappropriate pauses different between groups; also the intersentential pauses before commas were different, since third grade children with poorer comprehension made more pauses before commas than the same group in sixth grade. This was seen by Miller and Schwanenflugel (2006) in a study with third grade children and by Chafe (1988) in his study with adults, where more skilled readers may not feel driven to mark every comma with a pause. Our results agree with those findings, since the group with poor reading comprehension, who are less skilled, made more pauses before commas than the group with good reading comprehension; besides, within the group with poor comprehension, third-grade children, younger and having lower reading skills, paused more often than children from sixth-grade.

Moreover, a correct prosody involves a proper melodic contour, suitable for every type of sentence. Particularly, in declarative and exclamatory sentences the pitch falls at the end of the sentence, while in the yes-no questions the pitch rises (Miller and Schwanenflugel, 2006). Our results showed that only the children with better reading comprehension made a final declination in declarative sentences. That was found by other authors that related a final declination pitch in declarative sentences in better readers (Ladd, 1984; Wichmann, 1994; Benjamin and Schwanenflugel, 2010). We also found differences in the total range of pitch in the interrogative sentences of third grade, since children with less reading comprehension had a bigger pitch range. We could think that these poor young readers exaggerate the pitch contour when faced with a question mark. It is already known that children are aware of the different linguistic marks, such as exclamatory signs or quotes (Schwanenflugel et al., 2015), as they modify the tone and intensity when they encounter them. It is not surprising, therefore, that in the early stages of learning to read they tend to exaggerate those prosodic features of certain linguistic marks.

To sum up, the current study provides information about the relationship between prosody and reading comprehension, which is a little-studied field, but of great interest to education, since one of the major problems encountered in the classroom is the low reading comprehension presented by students. Determining the direction of this relationship is still needed. However, we have seen that there are different prosodic features, such as pauses or intonation of declarative and interrogative sentences, which differ according to the levels of understanding of the subject.

## Conflict of Interest Statement

The authors declare that the research was conducted in the absence of any commercial or financial relationships that could be construed as a potential conflict of interest.
